# Supplementary material for: The transcriptome of Listeria monocytogenes during co-cultivation with cheese rind bacteria suggests adaptation by induction of ethanolamine and 1,2-propanediol catabolism pathway genes
Source: PLoS One. 2020 Jul 23;15(7):e0233945. doi: 10.1371/journal.pone.0233945 (PMC7377500; doi:10.1371/journal.pone.0233945)
Supplement: S3 Table — (PDF) [file pone.0233945.s003.pdf]

S3 Table. Prophage genes significantly upregulated after 72 h co-cultivation of *L. monocytogenes* 6179 on plates with *Psychrobacter* L7

Prophage	Total no. of prophage genes	No. of upregulated prophage DE genes*
lma	15	14
tRNA-Arg-TCT	60	29
tRNA-Arg-CCG	68	27
tRNA-Thr-GGT	63	26

*no downregulated DE genes were identified for the prophages under this condition
