# Supplementary material for: The transcriptome of Listeria monocytogenes during co-cultivation with cheese rind bacteria suggests adaptation by induction of ethanolamine and 1,2-propanediol catabolism pathway genes
Source: PLoS One. 2020 Jul 23;15(7):e0233945. doi: 10.1371/journal.pone.0233945 (PMC7377500; doi:10.1371/journal.pone.0233945)
Supplement: S4 Table — Values show the log2 fold change of gene expression for the respective condition. (PDF) [file pone.0233945.s004.pdf]

S4 Table. Selected differentially expressed genes showing consistent gene expression changes in multiple co-cultivation conditions. Values show the log2 fold change of gene expression for the respective condition.

L. monocytogenes 6179 Locus_tag	*L. monocytogenes* EGDe locus_tag	Gene	*L. monocytogenes* 6179 and *Psychrobacter* L7 2 h broth	*L. monocytogenes* 6179 and *Psychrobacter* L7 12 h broth	*L. monocytogenes* 6179 and *Psychrobacter* L7 24 h plate	*L. monocytogenes* 6179 and *Psychrobacter* L7 72 h plate	*L. monocytogenes* 6179 and *Brevibacterium* S111 2 h broth	*L. monocytogenes* 6179 and *Brevibacterium* S111 12 h broth	*L. monocytogenes* 6179 and *Brevibacterium* S111 72 h plate
LM6179_0492	lmo0202	*hly*	1.17	ns*	ns*	3.54	1.05	ns*	4.32
LM6179_2386	lmo1634	*lap*	0.80	0.97	8.41	5.00	ns*	0.98	2.01
LM6179_0655	lmo0355	*frdA*	ns*	1.92	3.76	ns*	ns*	1.03	ns*
LM6179_0048	lmo2636	*pplA*	ns*	ns*	2.15	-1.18	ns*	0.71	ns*
LM6179_0049	lmo2638	*ndh2*	ns*	0.84	ns*	-1.03	ns*	0.97	-1.35
LM6179_0129	lmo2715	*cydD*	ns*	1.86	ns*	2.70	ns*	0.89	ns*
LM6179_0130	lmo2716	*cydC*	ns*	1.96	ns*	ns*	ns*	1.23	ns*
LM6179_0131	lmo2717	*cydB*	ns*	1.63	ns*	ns*	ns*	1.08	ns*
LM6179_0132	lmo2718	*cydA*	ns*	0.98	ns*	ns*	ns*	0.58	ns*
LM6179_0292	lmo0013	*qoxA*	ns*	-2.04	-1.61	ns*	ns*	-0.89	ns*
LM6179_0293	lmo0014	*qoxB*	ns*	-1.42	ns*	ns*	ns*	ns*	ns*
LM6179_0294	lmo0015	*qoxC*	ns*	-1.11	ns*	ns*	ns*	ns*	ns*
LM6179_0295	lmo0016	*qoxD*	ns*	-1.12	ns*	ns*	ns*	ns*	ns*

*ns denotes that the gene was not DE in that condition
