# Supplementary material for: The transcriptome of Listeria monocytogenes during co-cultivation with cheese rind bacteria suggests adaptation by induction of ethanolamine and 1,2-propanediol catabolism pathway genes
Source: PLoS One. 2020 Jul 23;15(7):e0233945. doi: 10.1371/journal.pone.0233945 (PMC7377500; doi:10.1371/journal.pone.0233945)
Supplement: S2 Fig — Panels A. and B. correspond to plate co-cultivation replicates of L. monocytogenes 6179 and Psychrobacter L7 and their corresponding monoculture controls after 24 and 72 h incubation periods, respectively. Panels C. and D. correspond to plate co-cultivation replicates of L. monocytogenes 6179 and Brevibacterium S111 and their corresponding monoculture controls after 24 and 72 h incubation periods, respectively. (PDF) [file pone.0233945.s006.pdf]

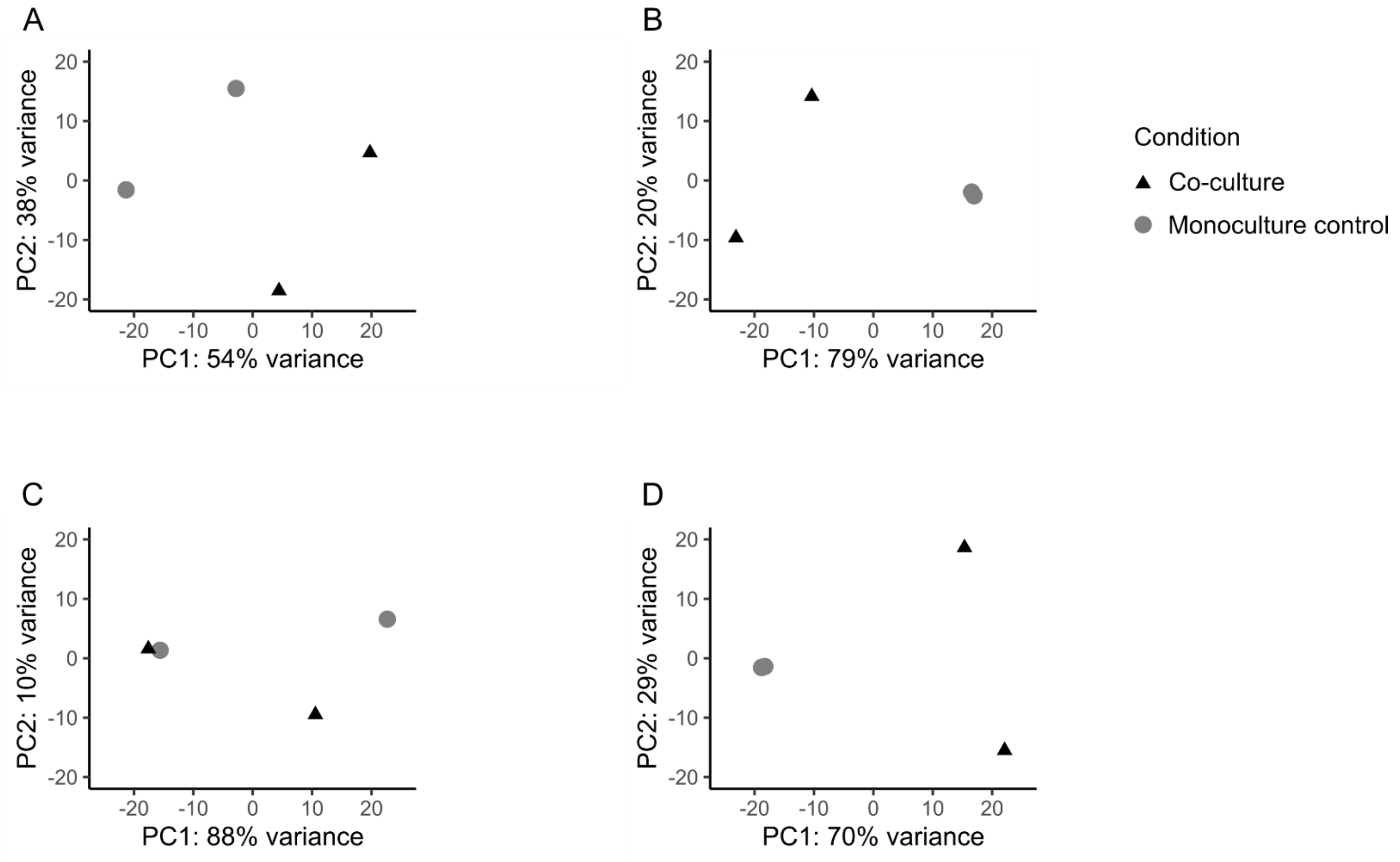

S2 Fig. Principal component analyses to visualize variance between *L. monocytogenes* 6179 transcriptome replicates of plate co-cultivations and the respective monoculture controls. Panels A. and B. correspond to plate co-cultivation replicates of *L. monocytogenes* 6179 and *Psychrobacter* L7 and their corresponding monoculture controls after 24 and 72 h incubation periods, respectively. Panels C. and D. correspond to plate co-cultivation replicates of *L. monocytogenes* 6179 and *Brevibacterium* S111 and their corresponding monoculture controls after 24 and 72 h incubation periods, respectively
