# Supplementary material for: The transcriptome of Listeria monocytogenes during co-cultivation with cheese rind bacteria suggests adaptation by induction of ethanolamine and 1,2-propanediol catabolism pathway genes
Source: PLoS One. 2020 Jul 23;15(7):e0233945. doi: 10.1371/journal.pone.0233945 (PMC7377500; doi:10.1371/journal.pone.0233945)
Supplement: Attachment — Submitted filename: Response to reviewer comments.pdf [file pone.0233945.s007.pdf]

Response to reviewer comments PONE-D-20-14512

Reviewer #1:

The work deals with the study of the transcriptome of L. monocytogens during its co-cultivation with microbes isolated from cheese rind and how the pathogen is adapted under different conditions of co-cultivation. The manuscript is well written. The methodology is clear for repetition and the experiments were logically designed. The statistical analysis, interpretation and discussion of the results were correct. According to authors' statement all the data are available without restriction.

The only comment is for Table 1 in which the number of predicted plasmids is three while the number given for the predicted plasmid contig size(s) in kbp are four. Please explain with a footnote.

Response: *There are three predicted plasmids in the strain, but one of them is predicted to consist of two contigs. As suggested, we have clarified this in a footnote.*
